# Iranian propolis efficiently inhibits growth of oral streptococci and cancer cell lines

**DOI:** 10.1186/s12906-019-2677-3

**Published:** 2019-10-11

**Authors:** Fariba Asgharpour, Ali Akbar Moghadamnia, Ebrahim Zabihi, Sohrab Kazemi, Amirmorteza Ebrahimzadeh Namvar, Hemmat Gholinia, Mina Motallebnejad, Hamid Reza Nouri

**Affiliations:** 1 Student Research Committee, Babol University of Medical Sciences, Babol, Iran; 2 Dental Materials Research Center, Health Research Institute, Babol University of Medical Sciences, Babol, Iran; 3 Department of Pharmacology, Faculty of Medicine, Babol University of Medical Sciences, Babol, Iran; 4 Neuroscience Research Center, Health Research Institute, Babol University of Medical Sciences, Babol, Iran; 5 Cellular and Molecular Biology Research Center, Health Research Institute, Babol University of Medical Sciences, Babol, Iran; 6 Department of Microbiology, Faculty of Medicine, Babol University of Medical Sciences, Babol, Iran; 7 Clinical Research Development Unit of Ayatollah Rohani Hospital, Babol University of Medical Sciences, Babol, Iran; 8 Oral Health Research Center, Health Research Institute, Babol University of Medical Sciences, Babol, Iran

**Keywords:** Anti-bacterial, Anti-biofilms, Propolis, Quercetin

## Abstract

**Background:**

Propolis is a natural bee product with a wide range of biological activities that are related to its chemical composition. The present study investigated the quantification of quercetin (Q) in Ardabil ethanol extract of propolis (AEEP), and then compared its anti-bacterial, anti- biofilm and cytotoxic effects on cancer and normal cell lines.

**Method:**

In the present study, the chemical composition of AEEP was determined through the high-performance liquid chromatography (HPLC). The AEEP and its main component, quercetin (Q), were evaluated in vitro against 57 oral streptococci by a broth micro-dilution method. The biofilm formation was assessed through the crystal violet staining and MTT assays. The impact of AEEP and Q anti-proliferative effect were evaluated on the fibroblast as normal and cancer cell lines (KB and A431).

**Results:**

The Q concentration in the composition of AEEP was 6.9% of all its components. The findings indicated that the AEEP and Q were efficient against the cariogenic bacteria and were able to inhibit the *S.mutans* biofilm adherence at a sub-MIC concentration. Moreover, electron micrographs indicated the inhibition of biofilms compared to control biofilms. In addition, the AEEP and Q indicated a dose-dependent cytotoxic effect on A431 and KB cell lines. On the contrary, they had no cytotoxic effect on fibroblast cells.

**Conclusion:**

The results indicated that the synergistic impact of main components of AEEP was related to the inhibition of the cancer cell proliferation, cariogenic bacteria and oral biofilm formation. It may play a promising role in the complementary medicine and, it is suggested to be used as food additives.

## Background

The propolis is a nontoxic natural resinous substance that is produced by honeybee from diverse plants containing several chemical compounds such as flavonoids, phenolic acids, and aromatic compounds [1, 2]. The ingredient of this compound depends on its geographic origin and other factors such as collection periods by honeybees [3]. Propolis has a wide range of biological functions such as anti-microbial, anti-viral, anti-fungal, anti-inflammatory, anti-oxidative, free-radical scavenging and anti-tumor properties [2–6]. The anti-microbial activity of propolis is considerable and it varies according to the flavonoid content [7]. The reduction of bacterial count in oral cavity effectively prevents the teeth decay [8]. *Streptococcus mutans*, a main acidogenic bacterium, is crucial in developing malignant cariogenesis [9]. Due to the synthesis of extracellular polysaccharides, mainly water-insoluble glucan, *S. mutans* can be colonized on the teeth surface and can initiate the plaque formation and dental caries [10]. Propolis can adhere to the teeth surface, and subsequently decrease the dental plaque development [11–13]. According to the emergence of multidrug resistant strains, the reduction and elimination of the pathogenic bacteria and also maintaining the normal flora are critical to prevent the teeth decay and oral diseases [14].

Recent studies indicate that ethanol extract of propolis (EEP) exerts potent anticancer activities against many tumor cells. The results revealed anticancer properties of propolis via various mechanisms such as the inhibition of cell proliferation and growth, induction of apoptosis, cell cycle arrest and induction of mitochondrial stress. Propolis contains a wide range of polyphenol and flavonoid content that strongly depends on the geographical areas, time and place of collection, climate, and the surrounding vegetation. This wide spectrum of different factors can affect the biological activity of propolis [15].

Quercetin (3,3`,4`,5,7-penta hydroxyl flavanone) is a flavonoid that can be found in different types of plants; and propolis is a one of the richest flavonoid source. Quercetin has a wide range of pharmacological properties such as the anti-inflammation and anti-oxidation; hence, it can be beneficial to the human health. Previous studies on bioactive effects of quercetin revealed that it could inhibit the growth of different microorganisms, especially gram-positive bacteria (*Bacillus subtilis*, *Micrococcus luteus*, *Staphylococcus aureus* and *Staphylococcus epidermidis)* and gram negative bacilli (*Escherichia coli* and *Pseudomonas aeruginosa*) [16–19]. Quercetin applied anti-proliferative and antitumor activities via various mechanisms such as cell cycle arrest during G0/G1 or G2/M phases of the cell cycle in leukemia, breast carcinoma and esophageal adenocarcinoma cells [20–22]. The present study aimed to investigate the quantification of quercetin (Q) in the Ardabil ethanol extract of propolis (AEEP), and then compared its anti-bacterial, anti-biofilm and cytotoxic effects on cancer and normal cell lines with AEEP.

## Methods

### Preparation of Ethanolic extract of propolis

Parts of crude propolis produced by *Apis mellifera* bees were collected in the spring of 2016 from Ardabil in Northern of Iran. 2 g of propolis dissolved with 25 ml ethanol and shacked for 2 days at room temperature. Then, it was filtered and kept in the dark place. A rotary vacuum evaporator used for ethanol removal. The ethanol extract of propolis had a brown color and extracts stored at − 20 °C.

### High-performance liquid chromatography (HPLC) analysis

The propolis sample was analysis by HPLC consisted a chromatograph equipped with a UV detector (Knauer, Germany) and a normal-phase silica column (250 mm × 4 mm, 5 μm particle diameter, Eurospher C18m, Altmann Analytik, Germany) [23]. Quercetin was used as standard. The mobile phase was a mixture of 20% acetonitrile (solvent A) and 0.2% formic acids (solvent B) which were previously degassed and filtrated. The analysis started for 0 to 7 min and increased to 60% for 7–10 min, was kept a 90% for 10–12 min. The column temperature was set at 25 °C with a constant 0.8 ml/min flow. 20 μl of the quercetin as standard and sample solution was injection and the chromatograms were integrated at 270 nm.

### Bacterial strains

The plaque samples were collected with swab from the tooth surface in different age groups that referred to Department of Oral and Maxillofacial Medicine at Babol University of Medical Sciences, in 2017. The swabs were placed in a thioglycolate broth medium (Merck, Germany). Oral streptococci were isolated by Blood agar, MSB agar (mitis salivarius-bacitracin) and TYCSB agar (trypticase yeast extract cysteine sucrose with bacitracin). The plates were incubated in a jar (5% CO2) for 24 h at 37 °C. Identification of oral streptococci was performed according to standard methods [24], included colony morphology, gram positive cocci, record haemolytic reactions, catalase test, fermentation of different carbohydrates and finally confirmed with Microgen Strep ID. *Streptococcus mutans* (ATCC 35668), *Streptococcus sobrinus* (ATCC 27607) and *Streptococcus penumoniae* (ATCC 49619) were used as control species.

### Minimum inhibitory concentration (MIC)

A broth microdilution method was used to determine MIC according to CLSI broth microdilution method [24]. First, bacterial suspension (10^5^ CFU ml^− 1^) was inoculated into CAMHB (Merck, Germany) and dispensed at in 96-well (0.2 ml) microtiter plates. Two-fold serial dilutions of AEEP and Q were prepared and transferred to each well of the original extract in DMSO with final concentration from 1.56 to 1000 μg/ml. Final concentration of DMSO as solvent was 1%. The number of wells in each plate were allocated to negative control, a sterility control, and a control for the solvent (DMSO), the antibiotic penicillin tested as positive controls. Plates were anaerobically incubated for 24 h at 37 °C. MIC was determined as the lowest concentration of samples that had no macroscopically visible growth. Minimum bactericidal concentration (MBC) was determined by sub-culturing of three previous wells on Mueller Hinton agar and incubating for 24 h. The lowest concentration of samples with no bacterial growth (99% inhibition) was reported as MBC.

### Determination of anti-biofilms activity

The anti-adhesion propertie of AEEP and Q on *Streptococcus mutans* (ATCC 35668) was tested in a microtitre plate biofilm assay, with some modifications [25]. Isolate was cultured in 5 ml of BHI under anaerobic conditions at 37 °C for 48 h. To 100 μl BHI with 2% sucrose (w/v) containing sub-MIC of samples in each well added 100 μl of a standardized (5 × 10^5^ CFU ml^− 1^) of isolate. The control well was containing only BHI/sucrose (2% w/v). After incubating the plates in anaerobic conditions at 37 °C for 24 h, the plates were assessed by stain with crystal violet and the biofilms metabolic activity (MTT assay).

### Crystal violet staining assay

The following 24 h incubation, loosely attached cells were removed by three times washing of wells with PBS. After air-drying; the plates incubated in an oven at 60 °C for 45 min. Then, 100 μl of crystal violet 1% (w/v) was added to each well and were placed at room temperature for 15 min. The wells were washed 3–4 times with sterile distilled water to remove unabsorbed stain. Biofilm formation was evaluated by adding 125 μl of %95 ethanol to destain the wells. The absorbance at 590 nm was determined using a microplate reader (Rayto, RT- 2100C, Chinese). The percentage of inhibition was calculated for each concentration of the samples by the following formula: [(OD control- OD sample)/ OD control] × 100.

### MTT assay

MTT was dissolved in PBS to obtain a concentration of 5 mg/ml. Following remove and dried plates, the wells was filled with 100 μl of MTT solution and incubated for 3 h at 37 °C. The produced formazan was dissolved with add 150 μl of DMSO. The Optical density (OD) of each well was read by a microplate reader (Rayto, RT- 2100C, Chinese) at 570 nm. The percentage of inhibition were calculated by the following formula: [(OD control- OD sample)/ OD control] × 100.

### Scanning electron microscopy

For SEM analysis, the wells were washed with PBS and then fixed with a 4% glutaraldehyde solution for 24 h. The biofilms were dehydrated using a series of ethanol (50, 70, 90, and 100%) and dried for 24 h. The specimens sputter was coated with gold-palladium. The samples were analyzed by scanning electron microscopy (SEM; SNE- 4500 M, SEC CO., LTD, Suwon, Korea) [26].

### Cell cultures and cytotoxicity analysis

The cytotoxic effect of AEEP and Q was tested on normal human fibroblast and cancer cell lines including the mouth epidermoid carcinoma (KB) and skin squamous cell carcinoma (A431). KB and A431 cell lines was obtained from the National Cell Bank of Iran, Pasteur Institute (Tehran, Iran) and was cultured in RPMI 1640 containing L-glutamin and supplemented with 10% Fetal calf serum (FBS), and 1% PenStrep (penicillin G 100 IU/ml, streptomycin 100 μg/ml). Fibroblast was isolated as previously described. Briefly, fibroblast cells were isolated from human 1–3 months newborn foreskins that underwent routine circumcision in Amirkola Children Hospital, Babol/ Iran. The Ethics Committee of Babol University of Medical Science previously approved isolation of fibroblast [27]. Fibroblast cells were cultured in Dulbecco’s Modified Eagle Medium (DMEM); (Biowest, USA), 10% Fetal bovine serum (FBS) and 1% L-glutamine 2 mM with penicillin (100 U/ ml) and streptomycin (100 μg/ ml). The cell lines were grown as monolayers in 25 cm2 cell culture flasks at 37 °C in a 5% CO2 humidified atmosphere. Cells were treated with 0 up to 200 μg/ml of AEEP and Q. Each concentration was tested in triplicates along with the control group.

The cytotoxic effect was measured using the MTT assay after 48 h incubation with AEEP and Q. To determine the cell viability, 50 μl of MTT solution in PBS (5 mg/ml) was added cells were incubated for an extra time 4 h. Then, the insoluble formazan product was dissolved in 150 μl DMSO, and then the OD was measured using a microplate reader (Rayto, RT- 2100C, Chinese) at 540 nm [25]. The obtained OD from the control group was considered as 100% viability.

### Statistical analysis

Data were analyzed using GraphPad Prism v 6.07 (GraphPad Software Inc., La Jolla, CA, USA). Results were expressed as the mean ± SD. Comparisons between groups was performed via t-student tests and, the one-way ANOVA. *P* < 0.05 was considered statistically significant.

## Results

### HPLC analysis

**Fig. 1:**
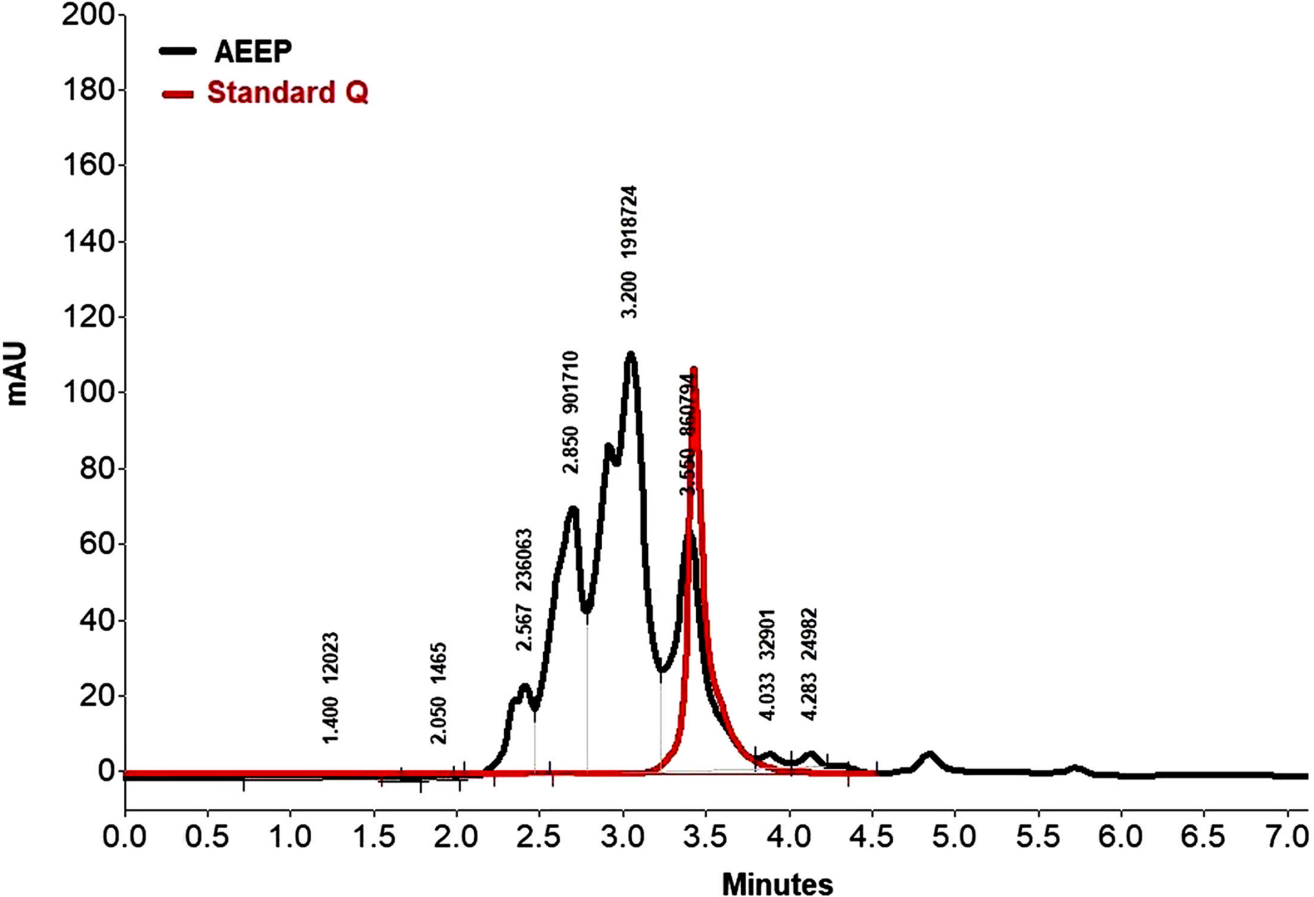
HPLC analysis of quercetin (red graph) and ethanolic extract of propolis (AEEP) that represented in black color

Based on calibration curves of the HPLC analysis, which was evaluated for standard Q, the concentration of this bioactive compound was determined in propolis. For quantification, acetonitrile and formic acids were used as the mobile phase. Identified and quantified quercetin was performed on the basis of the spectrum correlation with standard quercetin at 270 nm (Fig. 1). The Q concentration in the the composition of AEEP was 6.9% of all its total components.

### Antibacterial assay

Oral streptococci (*n* = 57), which was obtained from the culture included *S. salivarius* (*n* = 20), *S. mutans* (*n* = 20), *Streptococcus oralis* (*n* = 3), *Streptococcus mitis* biovar 1(*n* = 4), *Streptococcus uberis* (*n* = 5), *Streptococcus bovis* (*n* = 3), *Steptococcus equinus* (*n* = 1) and *Streptococcus parasanguinis* (*n* = 1).

### Minimum inhibitory concentration (MIC)

Table 1 presents MIC values for AEEP and Q against 57 oral streptococci and standard strains. The lowest MIC value belonged to *S. Salivarius*, which was treated with AEEP, and the highest MIC belonged to *Streptococcus penumoniae* (ATCC 49619) treated with Q*.* AEEP inhibited the growth of oral streptococci with MIC values from 3.12 to 100 μg/ ml. Penicillin inhibited oral streptococci with MIC values of 0.078–20 μg/ml. In most of isolates, MBC values of samples were 1 to 5 times higher than MIC values.

**Table 1:** The Minimum Inhibitory Concentration (MIC) values of samples against oral Streptococci (values in μg/ml)

Oral streptococci	AEEP		Quercetin		Penicillin	
	MIC	MBC	MIC	MBC	MIC	MBC
*Streptococcus salivarius* (20)	3.12–25	50–100	50–100	200–400	0.078–2.5	0.31–5
*Streptococcus mutans* (20)	6.25–25	50–100	50–200	100–400	0.078–0.31	0.31–0.62
*Streptococcus mitis biovar1*(4)	6.25–12.5	50	25–100	200	0.62–1.25	1.25–2.5
*Streptococcus parasanguinis*(1)	25	200	100	400	0.31	0.62
*Streptococcus uberis* (5)	25	100	100–200	200–400	1.25–2.5	2.5–5
*Streptococcus bovis* (3)	12.5	50	100	200	1.25–2.5	2.5–5
*Streptococcus equinus* (1)	12.5	100	100	200	2.5	5
*Streptococcus oralis* (3)	25	50	50–100	200	5–10	10
*Streptococcus mutans* ATCC 35668 *Streptococcus*	12.5	100	50	200	2.5	10
*sobrinus* ATCC 27607	25	100	100	200	2.5	10
*Streptococcus penumoniae* ATCC 49619	100	200	200	400	20	80

### Biofilm formation by *Streptococcus mutans*

**Fig. 2:**
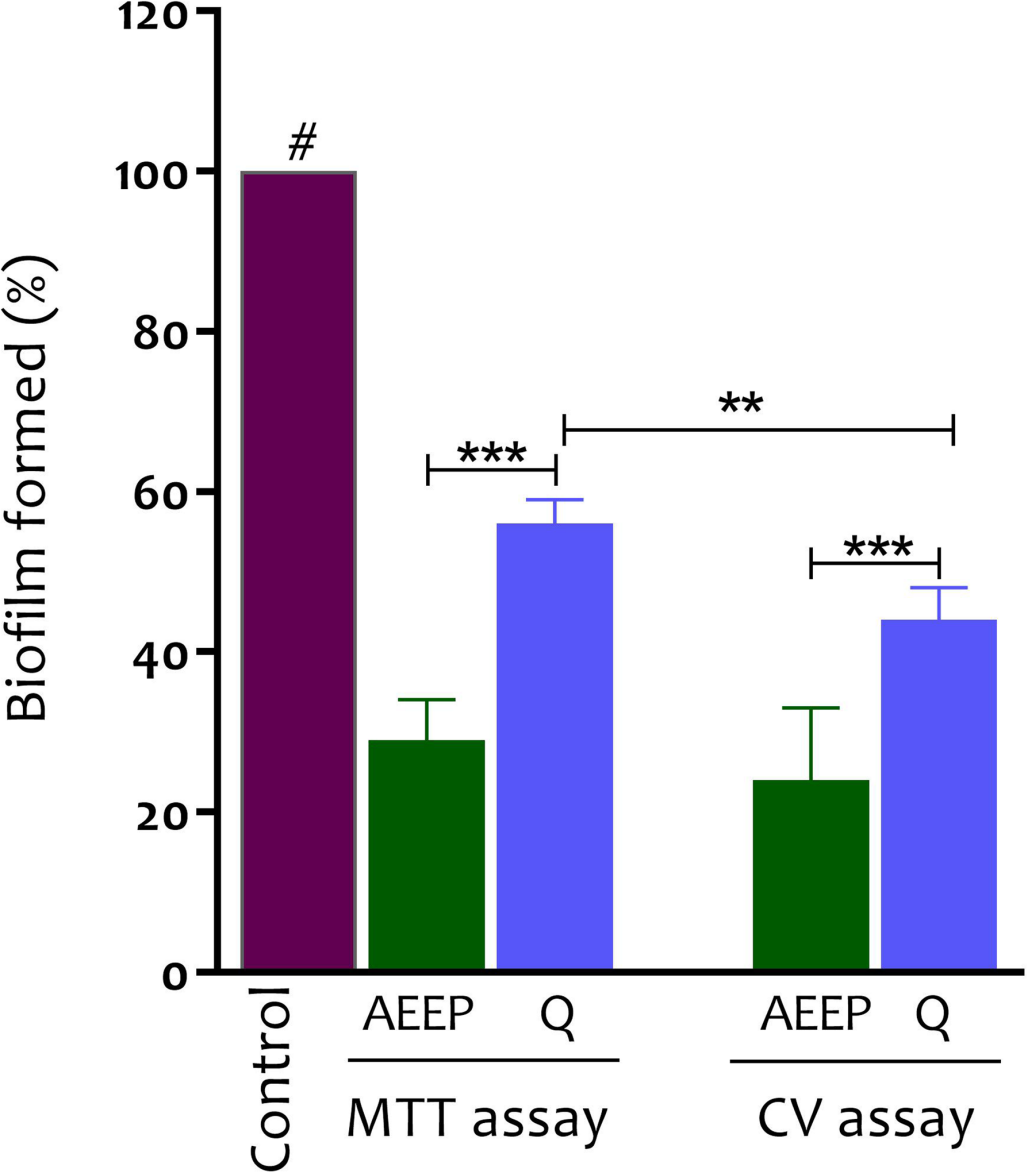
Effect of AEEP (6 μg/ml) and Q (20 μg/ml) on the biofilm formation at 24 h of growth phase of *Streptococcus mutans* (ATCC 35668) at sub-MIC level. The data represent an average of triplicate experiments ± SD (*n* = 3) and ***p < 0.001 and **p < 0.01 indicated in compare between samples. **#** The all treatments had significant difference in compare with the untreated control (*p* < 0.001)

**Fig. 3:**
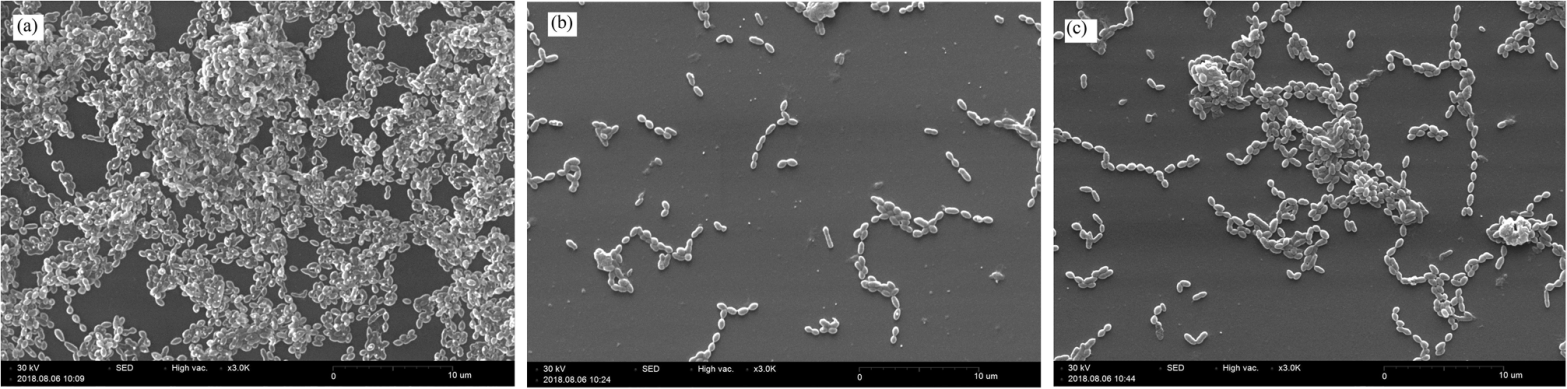
The scanning electron micrograph of *Streptococcus mutans* (ATCC 35668) biofilm formed after 24 h of incubation. **a** Control and in the presence of sub- MIC levels of **b** AEEP (6 μg/ml), **c** Q (20 μg/ml)

Figure 2 indicted the anti- biofilm effect of AEEP and Q at 24 h of growth phases of biofilm in CV and MTT methods. The percentage of adherent cells at concentration of sub-MIC of treatments significantly decreased at 24 h of the biofilm growth (*p* < 0.001) compared to control-biofilms. The maximum reduction in biofilm formation was estimated at 76 and 56% in CV method and 71 and 44% in MTT method in the presence of AEEP (6 μg/ml) and Q (20 μg/ml) respectively compared to the control. According to these findings, the most significant reduction was observed by AEEP. On the other hand, there was a significant difference between CV and MTT methods in the presence of Q. As shown in the Fig. 3, there are clear effects of AEEP and Q on the activity of *S. mutans* by scanning the electron microscopy. The control sample (Fig. 3a), showed biofilm formation after 24 h, while the number of cells decreased in the treated wells in sub-MIC (Fig. 3b and c).

### AEEP and Q had cytotoxic effects on A431 and KB, but not on fibroblast cells

**Fig. 4:**
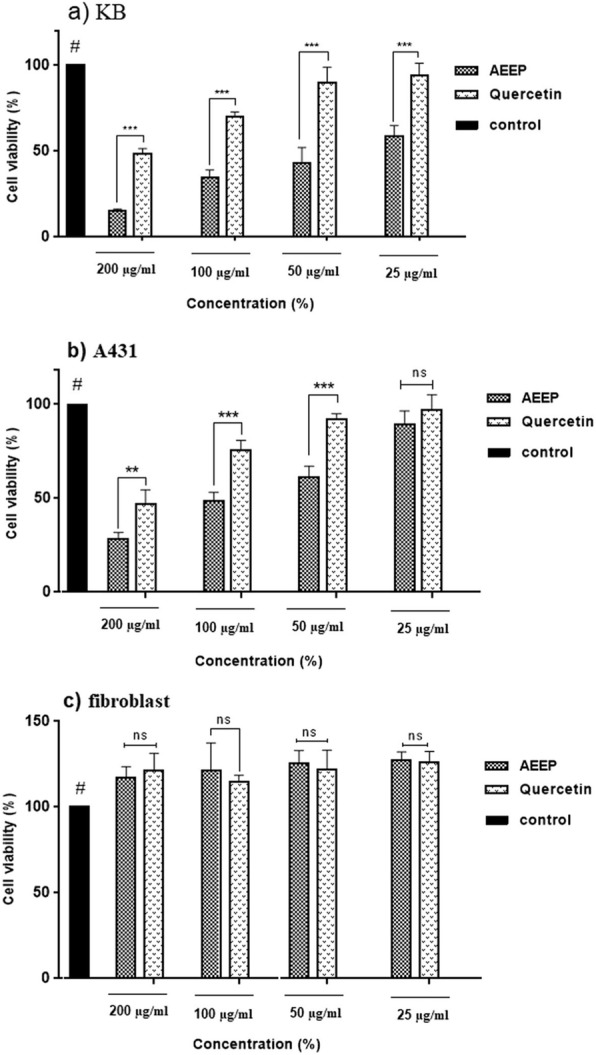
The percentage of cell viability by MTT exclusion on Mouth epidermoid carcinoma cell (KB) cells (**a**), skin squamous cell carcinoma (A431) cells (**b**) and fibroblast cell lines (**c**). Data are expressed as mean ± SD (*n* = 3). ***p < 0.001 and ** *p* < 0.01 indicated in compare between samples. **#** There was no significant difference between control with Q 50 μg/ml and Q 25 μg/ml in KB, 50 μg/ml and 25 μg/ml of AEEP and Q in A431 and the all concentrations of AEEP and Q in fibroblast cell lines (*p* < 0.05)

Figure 4 shows results of the cell incubation in the presence of 25–200 μg/ml of AEEP and Q after 48 h. AEEP and Q had cytotoxic effects on A431 and KB cell lines in a dose-dependent manner. IC_50_ values of AEEP and Q on KB cell line were 40 ± 8.9 μg/ml and 195 ± 14.9 μg/ml respectively after 48 h of incubation. The significant difference was observed between control and different concentrations of AEEP and Q (*p* < 0.001). On the contrary, 25 and 50 μg/ml of Q showed no significant difference with the control. As shown in Fig. 4a, a significant different was observed between AEEP and Q in each tested concentration. IC_50_ values of AEEP and Q were 98 μg/ml and 195 μg/ml respectively on the A431 cell line (Fig. 4b). Furthermore, a significant difference was observed between the control and 100 and 200 μg/ml of AEEP and Q (*p* < 0.001). In conclusion, the AEEP showed the highest cytotoxicity on targeted cancer cell lines. On the other hand, 25–200 μg/ml of both AEEP and Q tended to increase fibroblast cells count after 48 h, but there were no statistical significant difference (*p* > 0.05) with the control (Fig. 4c). The percentage of cell viability between AEEP and Q was not statistical significant in all tested doses (*p* > 0.05).

## Discussion

Propolis has a considerable variety of biological properties and therapeutic activity. It is relatively non-toxic and indicated anti-microbial activity against several bacteria, in particular, oral pathogens [28] and it is likely to directly affect the microorganisms (in vitro) and may activate mechanisms that are involved in killing microorganisms by stimulating the immune system (in vivo) [29]. Propolis has been taken into account in many studies in terms of dentistry to be beneficial in oral health including dental caries prevention, inhibiting the oral cancer growth, periodontal diseases, plaque formation and decreasing the incidence of oral mucositis after chemotherapy and it acts as the anti-inflammatory agent [2]. The biological activity of propolis is related to its origin such as the content of phenols and flavonoids. Flavonol quercetin (Q) is well-known in flavonoids. According to recent studies, Q indicates a wide range of pharmacological properties especially anti-cancer effects and anti-bacterial activity [30, 31]. In the present study, we examined effects of AEEP (containing 10.56 μg/mL of Q that was detected in HPLC analysis) and Q on inhibiting the growth of oral streptococci and studied their anti-tumor efficacy.

AEEP inhibited the growth of oral streptococci with MIC values ranging from 3.12 to 100 μg/ml. The obtained results were similar to the Tunisian propolis on oral streptococci with MIC values ranging from 2 to > 512 μg/ml [25], Brazilian propolis on dental caries ranging from 25 to 400 μg/ml [32]. Geopropolis (EEGP) also inhibited the growth of *S. mutans* UA 159 at concentrations lower than 50 μg/ml and reported that extracts were considered promising when MIC value was below 500 μg/ml; and this result was consistent with the present study [11].

The results indicated that Q inhibited the growth of streptococci strains at concentrations of 25 to 200 μg/ml, whereas up to 100 mg/ml of Q and its esters in a study by Gatto et al.; indicated no effect on tested microorganisms [31]. On the contrary, Woźnicka et al. indicated that Q with MIC values of 6.25 μg/ml was able to inhibit all strains; and NaQSA (sodium salt of quercetin-5í-sulfonic acid) reduced MIC values of *S. aureus* ATCC 29213 to 3.9 μg/ml and indicated that structures of drugs were related to their biological properties [33].

In most oral streptococci, glucans include extracellular thin layers that are produced in the presence of sucrose leading to the adhesion and biofilm formation of dental plaque. The formation of biofilms requires the initial binding of bacteria to a single surface and occurs in the subsequent bacterial population [34]. Propolis contains natural bioactive compounds and is effective in reducing oral biofilm bacteria [25]. In the present study, two methods were used to evaluate the anti-biofilm effects of AEEP and Q on *S. mutans* according to the MTT assay (a colorimetric assay for determining the cell metabolic activity) and the crystal violet assay (a simple method for staining attached cells). Both methods indicated good anti-biofilm activities of compounds that inhibited the biofilm formation. Chilean propolis affected the biofilm formation by *S. mutans* by an inhibition of 50% [9]. The EEPG inhibited the adherence of *S. mutans* growing cells at sub-MIC concentrations with a rate of 51% at 25 μg/ml [11]. On the contrary, Tunisian propolis inhibited the oral biofilm at a concentration of higher than MIC compared to other studies [25]. According to reports, Quercetin inhibited the anti-biofilm activity against bacterial pathogens such as *Pseudomonas aeruginosa* [16, 17], *Staphylococcus aureus* [18]*, Streptococci mutans* [26] and *Listeria monocytogenes* [19] indicating that Q had a potential of controlling the biofilm formation and may inhibit biofilm through specific mechanisms such as the initial adhesion or production of extracellular polymeric substances [19]. Therefore, the biofilm inhibitory effect of Q in the present study might be through similar mechanisms. Furthermore, Hasan et al. indicated that the maximum reduction in biofilm formation was at 24 h of the growth phases in the presence of Q; and the most significant reduction was seen by the combination of Q and Deoxynojirimycin compared to the control [26].

AEEP and Q inhibited the growth of KB and A431 cancer cells in a dose-dependent manner, but they had no effect on fibroblast cells compared to the control. Propolis solvent had no effects on viable cells; and the cytotoxic effects were solely due to propolis components. Due to reported side effects of surgery, radiotherapy and chemotherapy in the cancer treatment process, there has been increasing interest in the research on natural products, which were effective in the cancer prevention [30]. Propolis is a natural source with cytotoxic effects on different cell lines. In this regard, Tunisian propolis has a cytotoxic effect *on* Raw 264.7 cells, epithelial cell line (Hep-2), the respiratory epithelial cell line (A549), the intestinal epithelial cell line (HT-29) and normal human fibroblast-like fetal lung cell line (MRC-5) in a dose- and time-dependent procedure [25]. Moreover, the Brazilian propolis inhibited the growth of tumor cells and showed an anti-cancer potential [35]. The study on the Iranian propolis on the gastric cancer in rats revealed the chemo-protective effect on MNNG-initiated gastric cancer through inhibiting the cell proliferation and apoptosis induction [36]. Najafi et al. studied water extracts of the Iranian propolis (WEP) on cancer and normal cell lines. Furthermore, WEP comprised only the soluble part of propolis, but it was able to inhibit the growth of cancer cells and stimulate the growth of normal cells up to 60% compared with the control [37]. Effect of 25 to 800 μg/ml of the Iranian propolis extracts was evaluated on the viability of L929 fibroblast cells and did not show any cytotoxicity in ≤200 μg/ml; however, the viability was reduced to 20–50% at 400–800 μg/mL [38]. The results indicated that propolis of up to 200 μg/ml could stimulate the growth of normal cells. More studies showed that oxidative stress is a possible mechanism involved in apoptosis and necrosis induction. Increase level of reactive oxygen species (ROS) can affect cancer cells and lead to apoptosis and cell cycle arrest [39]. Accordingly, for evaluation of AEEP mechanism of action on ROS, we previously determined this mechanism on treated or untreated breast cancer cell line, MCF-7 via flow cytometry. Results shown that the levels of ROS increased significantly in MCF-7 cells in a dose-dependent manner compared with the control group. The observed results of ROS evaluation were accordance with cytotoxic activity on MCF-7 cells [40].

In previous studies on the impact of flavonoid quercetin on the oral squamous cell carcinoma (OSCC), it was found that Q inhibited the cell growth in a dose-dependent way and induced apoptosis via the NF-κB pathway and inhibited the cell proliferation through G1-phase arrest and mitochondrial mediated apoptosis [30]. Another study evaluated the role of quercetin in inhibiting the growth and inducing apoptosis on MCF-7 cells and indicated that when Q was protected by nanostructures was more effective on the inhibition of MCF-7 proliferation by blocking the cell cycle and promoting apoptosis [17].

The findings suggested that AEEP and Q might have a chemo-preventive potential or act as an adjuvant therapeutic agent for tumor cells. Despite the fact that a number of reports indicated that specific compounds in propolis were responsible for their bioactivity against different diseases, other un-identified compounds in propolis could synergistically affect the bioactivity especially the inhibition of cancer cells and support of normal cells.

## Conclusion

In summary, the obtained results indicated that AEEP and Q could efficiently inhibit oral streptococci through their antibacterial and cytotoxic effects on cancer cells lines, but it had no cytotoxic effects on normal cells. Since the North of Iran is reach in plants sources and local flora, which directly affect individual propolis components, it is highly recommended to look for mechanisms related to the antibacterial activity and inhibition of cancer cells by propolis and active ingredients in future studies.

## Data Availability

All data and materials of this work are available from the corresponding author on request.

## Abbreviations

AEEP: The ethanol extract of Iranian propolis from Ardabil origin
DMSO: Dimethyl sulfoxide
FBS: Fetal calf serum
HPLC: High-performance liquid chromatography
MBC: Minimum bactericidal concentration
MIC: Minimum inhibitory concentration
MSB: Mitis salivarius-bacitracin
MTT: 3-(4, 5-dimethylthiazolyl-2)-2, 5-diphenyltetrazolium bromide
PBS: Phosphate buffered saline
Q: Quercetin
SEM: Scanning electron microscopy
TYCSB: Trypticase yeast extract cysteine sucrose with bacitracin
